# Arsenoplatin-Ferritin Nanocage: Structure and Cytotoxicity

**DOI:** 10.3390/ijms22041874

**Published:** 2021-02-13

**Authors:** Giarita Ferraro, Alessandro Pratesi, Damiano Cirri, Paola Imbimbo, Daria Maria Monti, Luigi Messori, Antonello Merlino

**Affiliations:** 1 Department of Chemistry “Ugo Schiff”, University of Florence, Via Della Lastruccia, 3-13, Sesto Fiorentino, 50019 Florence, Italy; luigi.messori@unifi.it; 2 Department of Chemistry and Industrial Chemistry, University of Pisa, Via Giuseppe Moruzzi 13, 56124 Pisa, Italy; alessandro.pratesi@unipi.it (A.P.); damiano.cirri@unifi.it (D.C.); 3 Department of Chemical Sciences, University of Naples Federico II, Via Cinthia 21, 80126 Naples, Italy; paola.imbimbo@unina.it (P.I.); mdonti@unina.it (D.M.M.); antonello.merlino@unina.it (A.M.)

**Keywords:** Pt compounds, metal complexes, metallodrugs, As compounds, protein metalation, ferritin encapsulation, anti-cancer activity

## Abstract

Arsenoplatin-1 (AP-1), the prototype of a novel class of metallodrugs containing a PtAs(OH)_2_ core, was encapsulated within the apoferritin (AFt) nanocage. UV-Vis absorption spectroscopy and inductively coupled plasma-atomic emission spectroscopy measurements confirmed metallodrug encapsulation and allowed us to determine the average amount of AP-1 trapped inside the cage. The X-ray structure of AP-1-encapsulated AFt was solved at 1.50 Å. Diffraction data revealed that an AP-1 fragment coordinates the side chain of a His residue. The biological activity of AP-1-loaded AFt was comparatively tested on a few representative cancer and non-cancer cell lines. Even though the presence of the cage reduces the overall cytotoxicity of AP-1, it improves its selectivity towards cancer cells.

## 1. Introduction

Cisplatin, the first metallodrug with anticancer activity approved by FDA, entered the clinical practice at the end of the ’70s and, despite its severe side effects [1], is currently one of the most widely used drugs in anticancer therapy [2]. In this frame, the numerous adverse reactions associated with the use of cisplatin, prompted the scientific community to study many other Pt-based drugs as antitumor agents and to search for other anticancer compounds based on metals alternative to Pt, such as gold, ruthenium, iridium or arsenic [3]. In this respect, arsenic trioxide (As_2_O_3_), another inorganic drug approved by FDA, is employed for the treatment of acute promyelocytic leukemia (PML) [4]. Arsenous acid, the active form of As_2_O_3_, induces apoptosis through caspase activation and reactive oxygen species production [4,5]; moreover, it stimulates the proteasome, thus mediating the degradation of the PML-RARα oncoprotein, which plays a key role in PML development and is also able to target zinc finger and thiol rich proteins [6,7,8,9,10]. Small doses of As_2_O_3_ have minimal toxicity and are associated with prolonged patient’s survival [11].

Several studies combined cisplatin and As_2_O_3_ to take advantage of their antitumor activity. A significant synergy between these two drugs has been observed in several cancer cell lines [12,13]. Wang et al. found out an increased inhibition rate of As_2_O_3_ in human hepatocellular carcinoma when combined with cisplatin [12]. Zhang and co-workers obtained a similar result in multiple human ovarian cancer cell lines [13]. Furthermore, the cisplatin/As_2_O_3_ synergism was explored in small and non-small cell lung carcinoma [14]. According to Li and co-workers the two drugs cooperate to induce apoptosis probably through the induction of a caspase independent pathway [14].

**Figure 1:**
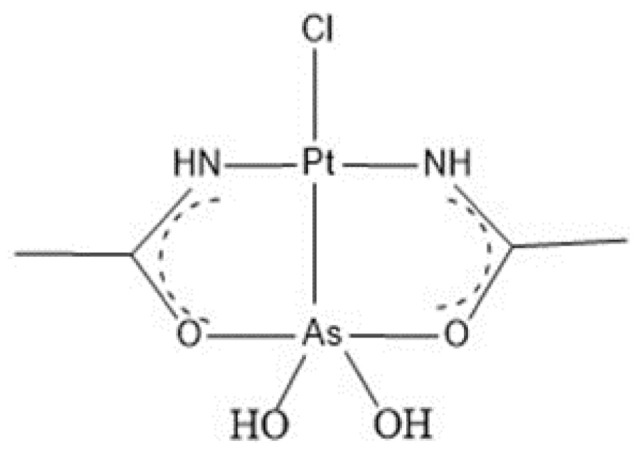
Schematic representation of AP-1.

Given these promising results, a new class of metal compounds containing a PtAs core has been designed [15]. Arsenoplatin-1 ([Pt(µ-NHC(CH_3_)O)_2_ClAs(OH)_2_]), also called AP-1 (Figure 1), is the most representative member of this class. This molecule is stable in solution and has chemical bonding, ligand substitution chemistry, and biological activities distinct from the parent compounds; moreover, it shows promising activity on drug-resistant cancer cell lines [16]. Indeed, AP-1 shows a higher cytotoxicity with respect to As_2_O_3_ and cisplatin on many cancer cell lines. It is able to bind proteins and DNA [17]; upon binding to DNA AP-1 releases the As(OH)_2_ moiety over time [17].

Thanks to the promising properties of AP-1, it is useful to evaluate if it is possible to improve its performances by encapsulation within a nanocarrier. A large variety of biomaterials can be used to selectively deliver drugs to their final targets. Protein nanocages attracted intense attention as drug delivery systems due to their high biocompatibility, high solubility, and ease of surface modification [18,19,20,21,22]. Among proteins, a special position is occupied by the ferritin superfamily [21] since it has been shown that these proteins can selectively deliver anticancer drugs to cancer cells [22,23,24]. As the ferritin (Ft) nanocage has already been used to encapsulate a variety of metallodrugs [25,26,27,28,29], we explore here whether it is an appropriate nanocarrier for AP-1.

## 2. Results

### 2.1. Preparation and Characterization of AP-1-Encapsulated Ferritin

**Figure 2:**
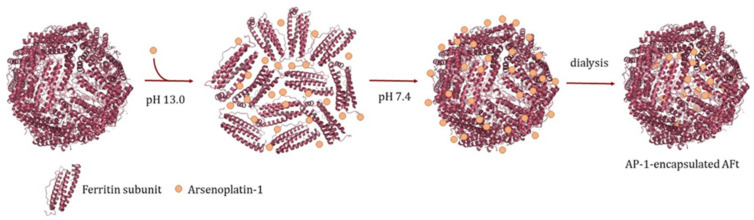
Schematic representation of the protocol used to encapsulate AP-1 within the AFt nanocage.

The AP-1-encapsulated horse spleen apo-ferritin (AFt) was prepared following the alkaline pH procedure previously reported for cisplatin-encapsulated AFt [30]. Briefly, the protein cage is disassembled at basic pH and then reassembled in the presence of a large amount of the metallodrug (a protein subunit to AP-1 molar ratio of 1:50 was used). In this way, the metal compound is trapped within the inner cavity of the cage (Figure 2). The same protocol was used to obtain an apo form of Ft to be used as control.

**Figure 3:**
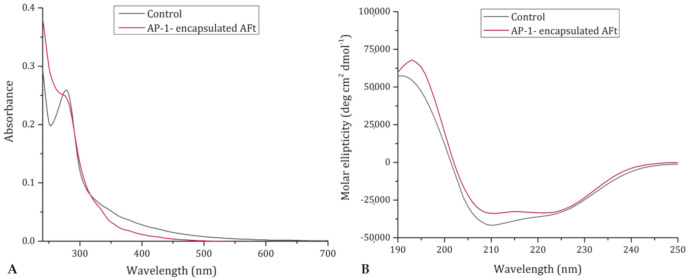
(**A**) UV–vis spectra of AP-1-encapsulated AFt (violet line) and of AFt used as control (control, black line). (**B**) Far-UV CD spectra of AFt (control, black line) and AP-1-encapsulated AFt (violet line).

To verify the formation of AP-1-encapsulated AFt and evaluate the amount of the drug that has been trapped within the protein cage, UV-vis spectroscopy and inductively coupled plasma-atomic emission spectroscopy (ICP-AES) were used. The UV-vis spectrum of AP-1-encapsulated AFt is reported in Figure 3A (violet line) superimposed to that of the control (black line). The absorption differences between 250 and 280 nm confirm that AP-1 was successfully encapsulated within the AFt nanocage. In turn, ICP-AES measurements indicate that AFt: AP-1 ratio in the sample is equal to 1:29. After extensive dialysis, AFt retains from 300 to 340 AP-1 molecules, depending on the preparation, achieving an average protein chain to metallodrug molar ratio of 1:14.

The protocol that was used to encapsulate AP-1 within the AFt nanocage can affect the overall structure of the protein, preventing its correct re-folding. Thus, the secondary structure content of the drug-loaded nanocomposite was characterized by collecting far-UV CD spectra of AP-1-encapsulated AFt (Figure 3B) and of the control. The far-UV CD spectrum of AP-1-encapsulated AFt shows a dominance of α-helix structure with broad negative minima at 208 and 222 nm (Figure 3B, violet line). The spectrum of the protein is almost superimposable to that of the control, suggesting that AFt can correctly reassemble in the presence of the drug.

The cage shape of AP-1-encapsulated AFt in solution was assessed by measuring its hydrodynamic diameter. Dynamic light scattering method provides information about the shape and effective diameter of the hydrated/solvated surface of molecules in aqueous solutions [31]. The AP-1 loaded nanocomposite presents a hydrodynamic radius R_H_ of ~21 nm, in good agreement with literature data for horse spleen ferritin, which has a R_H_ of ~23 nm [32]. The sample exhibits a good uniformity showing a polydispersity index of 0.321 [33]. Furthermore, zeta potential values of AP-1-encapsulated AFt and of negative control, i.e., the values of the electrostatic potential of protein surface respect to the bulk of the solution, were collected. Zeta potential was about −19.4 ± 1.4 mV for the AP-1 loaded nanocomposite and −18.4 ± 2.4 mV for the negative control at pH 7.4.

### 2.2. X-ray Structure of AP-1-Encapsulated AFt

**Figure 4:**
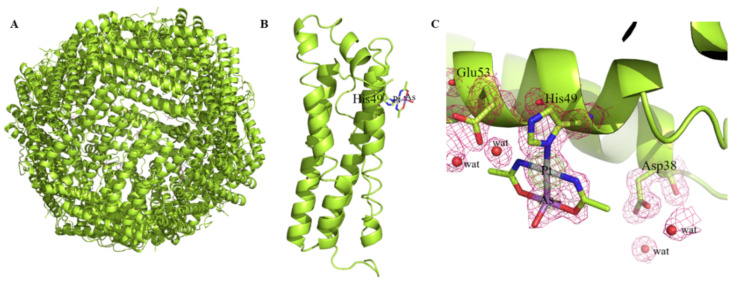
Cartoon representation of the overall structure of the cage (**A**) and of a single protein chain of AP-1-encapsulated AFt (**B**). The AP-1 binding site is also shown (**C**). 2Fo–Fc electron density map is contoured at 1σ (magenta) and 3σ (green).

AP-1-encapsulated AFt was then crystallized and X-ray diffraction data on these crystals were collected at 1.50 Å at high resolution at ESRF synchrotron in Grenoble, France. The overall structure of AP-1-encapsulated AFt, reported in Figure 4A, is very similar to that of the metal-free protein. Root mean square deviation between the carbon alpha atoms of each protein chain (Figure 4B) is as low as 0.13 Å. The structure shows that the Pt-As bond is retained in the adduct (Figure 4C). An AP-1 fragment (occupancy=0.40) binds the side chain of His49, upon releasing of the labile chloride ligand. The Pt(II) center coordinates the ND1 atom of the histidine side chain; the Pt-ND1 distance is 2.5 Å. The AP-1 fragment forms hydrogen bonds with the side chain of Glu53 and with solvent molecules. One of the OH groups bound to As interacts with the side chain of Arg52 and with a water molecule.

### 2.3. Drug Release from AP-1 Encapsulated AFt

The stability of AP-1-encapsulated AFt towards drug release was subsequently studied using dialysis. The drug release was evaluated at pH 7.4, 6.5, and 5.0, which simulated blood stream and normal physiological environment (pH 7.4), the mildly acidic environment in tumor tissues (pH 6.5), and the acidic lysosomes (pH 5.0), respectively [34,35]. ICP-AES measurements were conducted on the emission spectral line of As (188.980 nm) and the obtained data showed that an amount of AP-1 between 24 and 47% is released in 24 h under the three different conditions. The amount of AP-1 that is released by the cage is higher at pH 5.0 (47%) and at pH 6.5 (35%) than at pH 7.4 (24%). Indeed, the dialysis buffer at pH 5.0 contains an amount of AP-1 that is almost the double than that of the dialysis buffer at pH 7.4.

### 2.4. Cytotoxicity Studies

Finally, the cytotoxicity of AP-1-encapsulated AFt was tested on two cancer cell lines, i.e., murine BALB/c-3T3 fibroblasts transformed with SV40 virus (SVT2) and human epidermoid carcinoma (A431), and two non-cancer cell lines, i.e., immortalized murine BALB/c-3T3 fibroblasts (BALB/c-3T3) and immortalized human keratinocytes (HaCaT). Cells were incubated with increasing amounts of AP-1-encapsulated AFt and AP-1 for 48 h. At the end of incubation, cell viability was evaluated by MTT assay [36] and IC_50_ values of AP-1 and AP-1-encapsulated AFt, corresponding to the drug concentration able to inhibit cell growth by 50%, were determined (see Table 1). Values in Table suggest that, even if AP-1-encapsulated AFt was less cytotoxic with respect to AP-1, its selectivity towards cancer cells is increased, as the amount of AP-1 needed to kill cancer cells was at least the half than that needed to kill immortalized cells.

### 2.5. Cellular Uptake Studies

In order to evaluate if the difference in toxicity between AP-1 and AP-1-encapsulated AFt could be related to different amount of internalized AP-1 within the cells, uptake experiments have been carried out. One cancer cell line, A431 cells, and one immortalized cell line, HaCaT cells, have been treated with AP-1-encapsulated AFt at the concentration equal to IC_50_ (AP-1 concentration in the AP-1-encapsulated AFt = 10.91 μM) for 48 h. After incubation, the amount of AP-1 within the cells has been evaluated by ICP-AES. Results show that cancer cells have a higher amount of AP-1(30.9 ± 4.8 × 10^−9^ μg As/cell) than that found in healthy cells (8.7 ± 2.5 × 10^−9^ μg As/cell). These findings suggest that the selectivity of AP-1-encapsulated AFt could be due to a preferential entrance of the cage in cancer cells via AFt receptors, which are highly expressed in cancer cells [21].

## 3. Discussion

The antineoplastic properties of cisplatin are associated with adverse side effects and the occurrence of resistance mechanisms that greatly limit its efficacy. Today, research in the field of metals in medicine is focused on alternative metallodrugs that might allow to overcome these problems. Arsenoplatins are among the most promising metal compounds that have been developed as alternative to cisplatin in the last few years [15]. These molecules have a mechanism of action distinct from both cisplatin and As_2_O_3_ and are able to overcome resistance mechanisms [15]. The prototype of this class of compounds is AP-1 that is obtained in the reaction of cisplatin with As_2_O_3_ in acetonitrile-water mixture. AP-1 shows stronger activity than cisplatin in breast, leukemia, colon, and CNS cancer cell lines [16]. Since it has been demonstrated that AP-1 can bind proteins [17], here we have evaluated its encapsulation within the AFt nanocage, which has been demonstrated to target cancer cells [22,23,24]. The aim of the present paper was to study whether AP-1 encapsulation within a suitable nanocarrier could improve the performances of this already effective drug.

Thus, AP-1-encapsulated AFt has been produced and characterized; its anticancer activity against a couple of cancer cell lines has been evaluated. The presence of both Pt and As within the cage was confirmed by ICP-AES analysis. A ferritin cage can encapsulate 700 AP-1 molecules, retaining from 300 to 340 molecules after dialysis. This is not surprising as Ft can store up to 4500 atoms of Fe and it has been already observed that the same protein can encapsulate hundreds of molecules of other metallodrugs [37]. There are other examples of encapsulation of heterobimetallic compounds with AFt [38], but this is the first case where the compound retains its bimetallic nature upon loading within the protein cage.

The encapsulation of AP-1 within AFt does not significantly affect the overall structure of the protein, as revealed by CD spectra, hydrodynamic diameter measurements and structural studies. The protein retains its secondary structure content and can reassemble in its typical spherical arrangement. The presence of the drug does not affect the electrostatic potential of the outer surface of AFt. In fact, the binding site is located on the inner surface of the cage. Zeta potential measurements further confirm that there are no AP-1 molecules bound to the external protein surface, since AP-1 encapsulated AFt and the negative control show a comparable electrostatic potential value.

Consequently, AP-1-encapsulated AFt retains the chemico-physical features of the native protein upon AP-1 encapsulation, and thus might specifically target cancer cells, as the native protein does. The obtained X-ray structure unveils the nature of the interactions of AP-1 with AFt. Upon reaction with the protein, an AP-1 fragment that has lost the chloride ligand binds the side chain of His49 retaining the Pt-As bond. The binding of AP-1 to a His side chain is in agreement with the results obtained in the structural studies of adducts formed upon reaction of AP-1 with the model proteins hen egg white lysozyme (HEWL) and bovine pancreatic ribonuclease [17] and in the adduct formed by an aggregated form of AP-1 with HEWL [39].

Since more than one molecule of AP-1 per AFt chain is present, analysis of the protein structure points out that there are a lot of free AP-1 molecules trapped within the bulk. Similar results were found for other metallodrug-encapsulated Ft systems [40,41,42]. These molecules are responsible for the biological activity of the nanocomposite. In fact, we believe that, inside the cell, the cage is broken and the AP-1 molecules that are in the bulk can be released. It is possible that this could happen in acidic environments. The amount of AP-1 that is released from the protein shell in 24 h has been evaluated under three different pHs. Data suggest that the nanocomposite is more stable at neutral than at acidic pH, in agreement with previous experiments [43].

A first evaluation of the cytotoxic activity of the nanocomposite has been carried out. The obtained IC_50_ values show that the encapsulation within AFt reduces the overall toxicity of AP-1. However, the presence of the cage improves the selectivity of AP-1 towards cancer cells. This result is associated with the finding that AP-1-encapsulated AFt preferentially enters cancer cells when compared to healthy cells, as suggested by uptake experiments. This could be due to the overexpression of receptors that are responsible for Ft endocytosis in cancer cells.

## 4. Materials and Methods

### 4.1. Preparation and Spectroscopic Characterization of AP-1-Encapsulated AFt

AP-1 was synthetized following the procedure previously described [15]. Horse spleen ferritin was purchased by Sigma Chemical CO (Merck Life Science, S.r.l., Milan, Italy) and used without further purifications.

UV−vis absorption spectra of AP-1-encapsulated AFt were recorded using a 0.1 cm optical path-length quartz cell on a JASCO V-560 UV−vis spectrophotometer in the range of 240–700 nm, using a protein concentration of 0.25 mg mL^−1^ in 10 mM sodium phosphate pH 7.4. Other experimental parameters were: bandwidth 2.0 nm, scanning speed 200 nm min^−1^, data pitch 1.0 nm.

Far UV CD spectra were recorded on a Jasco J-715 spectropolarimeter equipped with a Peltier thermostatic cell holder (Model PTC-348WI) in the range of 190−250 nm, using protein concentration of 0.05 mg mL^−1^ in 10 mM sodium phosphate pH 7.4 and a 0.1 cm path length quartz cells. Each spectrum was obtained averaging three scans and converting the signal to mean residue ellipticity in units of deg cm^2^ dmol^−1^. Other experimental settings were: scanning speed 50 nm min^−1^, bandwidth 2.0 nm, resolution 1.0 nm, sensitivity 50 mdeg, and response 4 s.

Hydrodynamic size and surface charge (zeta potential) of AP-1-encapsulated AFt were assessed by means of dynamic and electrophoretic light scattering using a Zetasizer Nano ZSP (Malvern Instruments Cambridge, UK). All the measurements were performed using 1.5 mg mL^−1^ protein solutions in 10 mM sodium phosphate buffer pH 7.4, previously filtered with 220 nm cut-off microfilters, and polystyrene Folded Capillary Zeta cells (Malvern Instruments). Each measurement was performed at 25 °C upon 30 s equilibration time. The average of three measurements at stationary level has been taken. All DLS curves were acquired with a scattering detection angle of 173°. The hydrodynamic radius of ferritin was calculated through the Stokes–Einstein equation. Zeta potential was calculated by applying the Smoluchowski model. Zeta potential data were collected for the negative control too using the same experimental conditions.

### 4.2. ICP-AES Measurements

The determination of metal concentration in the AP-1-loaded AFt nanocage was performed as previously reported [44] by using a Varian 720-ES inductively coupled plasma atomic emission spectrometer (ICP-AES) equipped with a CETAC U5000 AT+ ultrasonic nebulizer, in order to increase the method sensitivity. For the of AP-1-loaded AFt solutions, 200 µL of each sample were used. To evaluate AFt loading and the amount of the drug released at different pH values, AP-1-loaded AFt was dialyzed against milliQ water overnight. Ferritin was recovered from the dialysis membrane and divided in three aliquots. Each aliquot was dialyzed against a specific buffer: 0.010 M citrate phosphate buffer pH 5.0, 0.010 M sodium phosphate buffer pH 6.5 and 7.4. Then, 200 µL of each of the three solutions and 4 mL of each of the respective dialysis buffers were processed and analyzed by ICP-AES.

The samples were then transferred into PE vials and digested in a thermo-reactor at 80 °C for 8 h with 2 mL of aqua regia (HCl supra-pure grade and HNO_3_ supra-pure grade in a 3:1 ratio). Ultrapure water (≤18 MΩ) was added only to the vials containing the AP-1-loaded AFt solutions until a final volume of 6 mL. All the samples were spiked with 1 ppm of Ge used as an internal standard and analyzed. Calibration standards were prepared by gravimetric serial dilution from a commercial standard solution of Pt and As at 1000 mg L^−1^. The following wavelengths were used: 214.424 nm for Pt, 188.980 nm for As, and 209.426 nm for Ge. The operating conditions were optimized to obtain maximum signal intensity, and between each sample, a rinsed solution of HCl supra-pure grade and HNO_3_ supra-pure grade at a 3:1 ratio was used to avoid any “memory effect”.

### 4.3. Crystallization and X-ray Diffraction Data Collection

Crystals of AP-1-encapsulated AFt were grown by hanging-drop vapor diffusion technique at 298 K mixing the protein (5–10 mg mL^−1^) with equal volumes of a reservoir solution consisting of 0.600–0.800 M ammonium sulphate, 0.100 M Tris pH 7.4–7.7 and 0.050-0.060 M cadmium sulphate. Best crystals grow within 7–10 days.

X-ray data for AP-1-encapsulated AFt were collected at ESRF synchrotron, Grenoble, France, using λ = 0.9677 Å (beamline ID30-A3). The programs HKL2000 [45], Mosflm [46], and SCALA [47] have been used for data indexing, reduction, and scaling. Crystal data and data collection parameters are given in Table 2.

### 4.4. Structure Solution and Refinement

Molecular replacement with the model extracted from the PDB file 5ERK [30] (without waters and ligands) and the program Phaser [48] were used to solve the phase problem. Refinement was carried out using Refmac5 [49]. Wincoot [50] has been used for model building and visualization of the electron density maps. The possible presence of As and Pt atoms has been evaluated considering anomalous difference, 2Fo-Fc and Fo-Fc Fourier difference electron density maps and comparing these maps with those of the corresponding regions in the native protein structure [30] and in the structures of cisplatin- and carboplatin-encapsulated AFt [30,51]. Refinement statistics are reported in Table 2 Coordinates and structure factors, including anomalous data, have been deposited in the Protein Data Bank under the accession code 7BD7.

### 4.5. Cytotoxicity and Uptake Experiments

Murine Balb/c-3T3 fibroblast, SVT2 and human epidermoid A431 cells were obtained from ATCC. Immortalized human keratinocytes (HaCaT) were from Innoprot (Biscay, Spain).

Cells were cultured in Dulbecco’s modified Eagle’s medium (DMEM) (Sigma-Aldrich, St Louis, MO, USA), supplemented with 10% fetal bovine serum (HyClone), 2 mM L-glutamine and antibiotics (Sigma-Aldrich), under a 5% CO_2_ humidified atmosphere at 37 °C. For cytotoxic analyses, cells were seeded in 96-well plates at a density of 2.5 × 10^3^ cells per well. 24 h after seeding, increasing concentrations of AP-1 (2.6–108 µM) and AP-1-encapsulated AFt (0.05–2.6 µM) were added to the cells. After 48 h incubation, cell viability was assessed by the MTT (3-(4,5-dimethylthiazol-2-yl)-2,5-diphenyltetrazolium bromide) assay as previously reported [36].

For uptake experiments, 1 × 10^6^ cells of both immortalized and cancer cells were plated and treated with AP-1-encapsulated AFt at the concentration equal to IC_50_ (AP-1 concentration = 10.91 μM) for 48 h. At the end of incubation, cells were treated as described in Annunziata et al. [52]. Each cellular pellet was transferred in a PE vial and digested in a thermo-reactor at 80 °C for 8 h with 2 mL of aqua regia (HCl supra-pure grade and HNO_3_ supra-pure grade in a 3:1 ratio). After this time, ultrapure water (≤18 MΩ) was added to the vials until a final volume of 6 mL. The metal concentration within cells has been determined using ICP-AES tuned on the As spectral line and the wavelength for As (188.980 nm), as described on Section 4.2.

## 5. Conclusions

A well-characterized bimetallic compound of potential medicinal interest, AP-1, has been encapsulated within the ferritin nanocage. The drug-loaded nanocarrier has been investigated:

From an analytical point of view, defining the exact amount of metal that is within the cage, and the protein-metallodrug stoichiometry.

From a structural point of view, analyzing the secondary structure content of the nanocomposite and solving its X-ray structure. Data allowed highlighting the nature of the interactions of AP-1 with the nanocarrier. AP-1 binds the side chain of His49 upon releasing the Cl^−^ ligand. The binding does not affect the overall structure and the chemico-physical properties of the protein nanocage. There is a significant amount of metal compound in the bulk.

From a biological point of view, testing the cytotoxic effects of the nanocomposite, in comparison to the free drug, on representative cancer and non-cancer cell lines. AP-1-encapsulated AFt kills tumor cells at a concentration lower than that needed to kill non-cancer cells, but the specificity is higher. The amount of AP-1 in cancer cells is higher than that observed in healthy cells when cells are treated with the same amount of drug-loaded system.

Overall, these data suggest that ferritin nanocages can be used to encapsulate arsenoplatins improving their selectivity towards cancer cells.

## Figures and Tables

**Table 1:** IC_50_ values obtained after 48 h of incubation with AP-1 and AP-1-encapsulated AFt based on the concentration of AP-1 and AFt, for on BALB/c-3T3, SVT2, and A431 cell lines.

		48 h Incubation		
		AP-1 (μM)	AP-1-Encapsulated AFt (μM/Ft chain)	AP-1-Encapsulated AFt (μM/AP-1)
**Non cancer cell lines**	HaCaT	2.60 ± 0.26	0.38 ± 0.02	10.91 ± 0.73
	Balb/c-3T3	3.60 ± 0.10	0.95 ± 0.00	27.80 ± 0.14
**Cancer cell lines**	A431	4.04 ± 0.92	0.16 ± 0.01	4.66 ± 0.29
	SVT2	3.87 ± 1.01	0.40 ± 0.01	11.64 ± 0.29

**Table 2:** Data collection and refinement statistics for AP-1-encapsulated ferritin.

Data Collection Statistics	
X-ray source	Synchrotron
Wavelength	0.9677 Å
Space group	F432
Unit cell parameters a = b = c (Å)	180.91
Molecules per asymmetric unit	1
Observed reflections	357664 (18387)
Unique reflections	40897 (2028)
Resolution (Å)	41.50–1.50 (1.53–1.50)
Completeness (%)	100.0 (100.0)
Rmerge	0.113 (0.840)
Rpim	0.040 (0.293)
Rmeas	0.120 (0.891)
I/σ(I)	12.4 (2.6)
Multiplicity	8.7 (9.1)
CC_1/2_	0.999 (0.563)
**Refinement Statistics**	
Resolution (Å)	41.50–1.50
N° reflections in working set	38816
N° reflections in test set	2059
N° non-H atoms in the refinement	1659
R factor/Rfree (%)	0.167/0.185
Estimated occupancy of Pt	0.40
Estimated occupancy of As	0.40
B-factor overall (Å^2^)	17.10
B-factor of Pt (Å^2^)	27.98
B-factor of As (Å^2^)	37.03
Ramachandran values (%)	
Most favored/ Additional allowed	0
Generously allowed/ Disallowed	3
R.m.s.d. from ideality	
R.m.s.d. bonds (Å)	0.014
R.m.s.d. angles (°)	1.63

Values in parenthesis refer to highest resolution shell.

## Data Availability

Not applicable.

## Abbreviations

A431	Human epidermoid carcinoma
AFt	Apoferritin
AP-1	Arsenoplatin-1
CD	Circular Dichroism
Cisplatin	*cis*-Pt(NH_3_)_2_Cl_2_
CNS	Central Nervous System
DNA	Deoxyribonucleic acid
FDA	Food and Drug Administration
Ft	Ferritin
HEWL	Hen Egg White Lysozyme
ICP-AES	Inductively Coupled Plasma-Optical Emission Spectrometry
MTT	3-(4,5-dimethylthiazol-2-yl)-2,5-diphenyltetrazolium bromide
PDB	Protein Data Bank
PML	Acute Promyelocytic Leukemia
UV	Ultraviolet
UV-vis	Ultraviolet-visible
